# Numerical Simulation of Light to Heat Conversion by Plasmonic Nanoheaters

**DOI:** 10.1021/acs.nanolett.4c04872

**Published:** 2024-12-19

**Authors:** María C. Nevárez Martínez, Dominik Kreft, Maciej Grzegorczyk, Sebastian Mahlik, Magdalena Narajczyk, Adriana Zaleska-Medynska, Demosthenes P. Morales, Jennifer A. Hollingsworth, James H. Werner

**Affiliations:** † Department of Environmental Technology, Faculty of Chemistry, University of Gdańsk, Wita Stwosza 63, 80-308 Gdańsk, Poland; ‡ Faculty of Mechanical Engineering and Ship Technology, Institute of Naval Architecture, Gdańsk University of Technology, Gabriela Narutowicza 11/12, 80-233 Gdańsk, Poland; § Faculty of Mathematics, Physics, and Informatics, Institute of Experimental Physics, University of Gdańsk, Wita Stwosza 57, 80-308 Gdańsk, Poland; ∥ Bioimaging Laboratory, Faculty of Biology, University of Gdańsk, Wita Stwosza 59, 80-308 Gdańsk, Poland; ⊥ Center for Integrated Nanotechnologies, Los Alamos National Laboratory, Los Alamos, New Mexico 87545, United States of America

**Keywords:** photothermal conversion efficiency, hanging droplet, Roper method, simulation, Wang method, gold nanorods

## Abstract

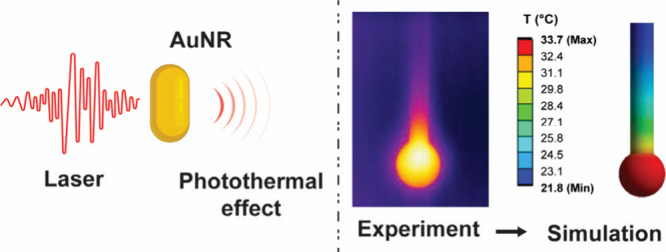

Plasmonic nanoparticles are widely recognized as photothermal conversion agents, i.e., nanotransducers or nanoheaters. Translation of these materials into practical applications requires quantitative analyses of their photothermal conversion efficiencies (η). However, the value of η obtained for different materials is dramatically influenced by the experimental setup and method of calculation. Here, we evaluate the most common methods for estimating η (Roper’s and Wang’s) and compare these with numerical estimates using the simulation software ANSYS. Experiments were performed with colloidal gold nanorod solutions suspended in a hanging droplet irradiated by an 808 nm diode laser and monitored by a thermal camera. The ANSYS simulations accounted for both heating and evaporation, providing η values consistent with the Wang method but higher than the Roper approach. This study details methods for estimating the photothermal efficiency and finds ANSYS to be a robust tool where experimental constraints complicate traditional methods.

Photothermal conversion mediated by plasmonic nanoparticles is an emerging technology in biomedical and commercial applications such as cancer therapy,^1−3^ pathogen deactivation,^4^ and decontamination systems.^5^ Photothermal conversion involves heating nanoparticles by irradiating them with light at a resonant frequency to cause electron oscillations, resulting in a series of decay pathways that ultimately transfer heat from the nanoparticle to the surrounding medium.^6−8^ The best means to calculating the efficiency of this process remains a debate. The photothermal conversion efficiency, η, strongly depends on the experimental setup, including the mass and geometry of the system, solution stirring, and how the temperature was recorded.^9^ As such, it is important to characterize both the experimental and compuational methods used to calculate η when evaluating plasmonic nanomaterials for their potential efficacy as nanotransducers or nanoheaters.

The most used methodologies for calculating η, namely Roper’s^10^ and Wang’s,^11^ differ in key aspects: Roper’s original setup consisted in a sealed quartz cuvette, while Wang’s consisted of an open quartz cuvette, with stirring, covered with plastic foam to avoid heat loss. A comprehensive analysis of these approaches can be found in greater detail in Paściak et al.^9^ Both systems included a relatively large mass that is not completely accounted for–the cuvette. A simplification presented by Meyer et al.^12^ considered only the mass of the cuvette in contact with the sample, which corresponded to 83% of the total cuvette mass. The authors indicated that if the full cuvette mass had been considered, efficiencies larger than 100% would have been computed.

Conveniently, Wang’s protocol^11^ saves us from assuming an effective mass–understood as the mass that effectively participates in heat transfer–by estimating it using an electrical resistance. The authors reported the effective cuvette mass to be less than 20% of the entire cuvette mass (standard quartz cuvette of 10 mm × 10 mm × 40 mm dimensions, without sample). While promising, it becomes necessary to do an effective mass calibration for each experiment, which can be time-consuming. This process can be avoided through Richardson’s^13^ hanging droplet setup, where a small sample droplet is carefully produced at the tip of a syringe. However, this strategy has limitations, such as potential droplet evaporation and difficulty positioning a thermocouple within a small-volume system. Carrying out the experiment in a humidity chamber has been suggested by Paściak et al.,^9^ which kept the evaporation (droplet shrinkage) at ∼9% during a 2 min irradiation experiment.

It is noteworthy that neither Roper’s nor Wang’s methods included evaporation in their heat balance equations. In our experience, evaporation happens during the measurement, even in quartz cuvettes, as small drops of condensate are observed on the internal cuvette walls (see Figure S1). The origin of this condensate comes from the resonant light absorption and ultrafast heat conversion, wherein the thin solvent layer surrounding nanoparticles is heated locally to temperatures higher than the boiling point and vaporizes creating nanobubbles that form a vapor shell. Under continuous light irradiation, the vapor shell grows or coalesces promoting the migration of steam bubbles toward the solvent-air interface. The vapor shell surrounding the nanoparticles acts as thermal barrier to reduce the thermal dissipation toward the bulk solution.^14^

Due to limitations with exisiting methods of estimating η, we leveraged advances in computer modeling, employing the ANSYS software (ANSYS Inc., Canonsburg, PA, USA)^15^ to explore numerical methods of determining η. ANSYS facilitates the simulation of thermodynamic phenomena using a finite element method, which leads to a reduction in costs and time. The software is also useful to validate numerical simulation assumptions with experimental results, with recent efforts in applying this tool to photothermal conversion phenomena. For example, Vence et al.^16^ studied the photothermal conversion of graphene oxide (GO) coated on 3D-printed polylactic acid (PLA) under 785 nm laser irradiation and used ANSYS to evaluate the optimum GO deposition, PLA thickness, and thermal conductivity of the probes. Moreover, the authors stated that ANSYS can be used to predict these parameters for different probe geometries. Filip et al.^17^ showed that ANSYS can be used to simplify the calculations of thermal properties derived from photothermal phenomena by applying a finite element method, which provides versatility and good agreement with experimental results. ANSYS also proved useful for simulating and modeling a photothermally actuated bent-beam microactuator, directing physical designs that performed similarly to the predictions.^18^

In this work, we employed a hanging-droplet scheme, similar to Richardson’s setup,^13^ but we measured droplet temperature and size (volume/mass) with a thermal camera (Figure S2). Rather than performing the experiments in a humidity chamber,^9^ to avoid sample evaporation, we preferred to account for evaporation in the heat transfer analysis.

In general, all methods for the quantification of η start from the energy balance equation (eq S1). Roper’s^10^ approach introduces the cooling time constant, τ_*c*_, to yield eq 1 (see Supporting Information for development of equations):
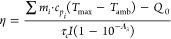
1Where ∑*m*_*i*_·*c*_*p_i_*_ is the sum of the products of mass times the heat capacity of each system component, *T*_max_ is the maximum temperature reached at steady state, *T*_amb_ is the ambient air temperature, *Q*_*O*_ is the transduced heat after irradiation of the solvent, which was determined to be negligible for distilled water and for phosphate buffered saline (PBS), *I* is the laser power (in W, measured with a power meter), *A*_λ_ is the absorbance of the sample at the laser irradiation wavelength λ (measured experimentally with a spectrophotometer or derived from the Beer–Lambert law).

In contrast to Roper’s method, Wang’s^11^ approach converts the heat balance equation into a descriptive function by introducing coefficients *a* and *b* (eq S7) and η is determined from eq 2.
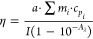
2*a* and *b* are numerically calculated by fitting the temperature curve to the data. For our system, *m* in eq 2 is the droplet/needle/water-in-needle mass and *c*_*p*_ is the average specific heat.

The method we developed uses the transient thermal module in the simulation software ANSYS. The droplet was modeled as a distilled water ellipsoid at the tip of a PTFE needle. We used colloidal gold nanorod (AuNR) solutions in two concentrations of Au^0^, 0.25 mM (∼50 μg mL^–1^) and 0.50 mM (∼100 μg mL^–1^), and also tested a PEGylated AuNRs (PEG-AuNRs) at 0.50 mM Au^0^ sample. We assumed that the influence of the AuNRs in solution is minimal on the density, viscosity, and thermal conductivity of the solvent, with these properties assumed to be identical for both water and PBS.

External conditions, which were kept constant throughout experiments, were considered as follows: the liquid in the syringe had the same temperature as ambient air (21.5 °C). The emissivity of the droplet was ε_*d*_ = 0.98 and for the needle ε_*n*_ = 0.97. The heat transfer coefficient between the needle and surrounding air was assumed to be 10 W m^–2^ K^–1^, while between the droplet and air, due to convective motion of water, was 50 W m^–2^ K^–1^.^19^ The thermal conductivity coefficient was assumed to be 0.6 W m^–1^ K^–1^, for distilled water, and 0.28 W m^–1^ K^–1^, for the PTFE needle.^20^ The heat flux, , resulted from the optimization carried out in ANSYS. It consisted in adjusting the heat flux value until the simulated temperature matched the experimental one. ANSYS calculations were based on the energy balance (eq S1) and the first law of Newtonian thermodynamics (eq 3).
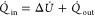
3Where  is the heat flux that was delivered to the sample in the form of laser light,  is the heat flux lost to the surroundings, and ΔU̇ represents the internal energy change of the system over time. ΔU̇ accounts for the temperature change as well as the fraction of the evaporated droplet. Thus, eq 3 develops into eq 4.
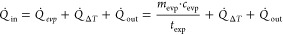
4Where *m*_evp_ is the evaporated mass during the experiment time *t*_exp_, and *c*_evp_ is the latent heat of evaporation for the droplet, which varied between 2442 kJ kg^–1^ and 2447 kJ kg^–1^,^21^ depending on the temperature reached during each experiment. Following these considerations, η can be calculated from eq 5.
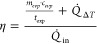
5

A flowchart describing the experimental analysis using ANSYS modeling is presented in Figure 1.

**Figure 1:**
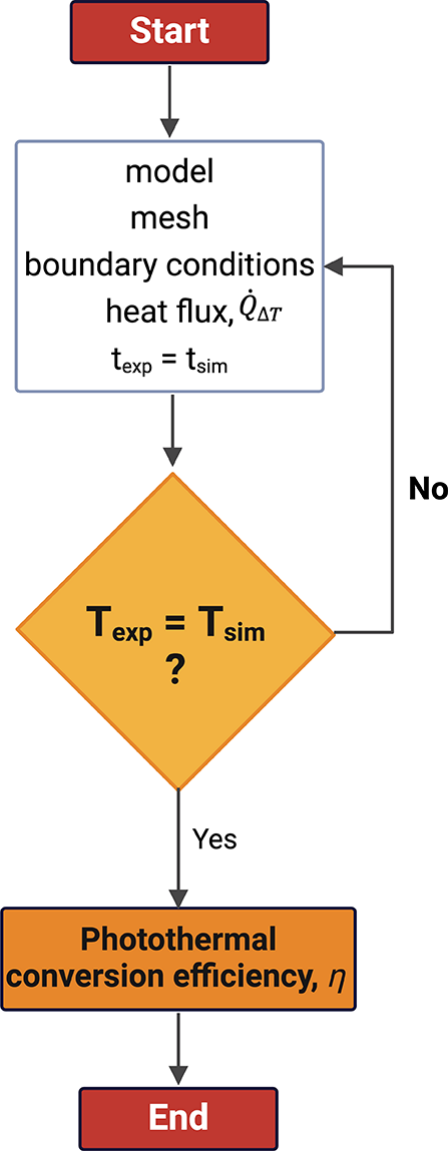
General flowchart for the numerical analysis using ANSYS. T is temperature and t is time, and subindexes exp and sim stand for experimental and simulated, respectively. Created in BioRender. Nevárez Martínez, M. (2024) https://BioRender.com/j85p746.

A simple scheme of the experimental setup is presented in Figure 2a. The laser illuminated the sample droplet from a 15 cm distance. The droplet was suspended from a PTFE needle attached to a syringe. A syringe pump system, with a stepper motor, controlled the syringe plunger. The total laser beam illuminated ∼60–80% of the droplet, which was considered in determining the incident laser power. Additionally, the absorbance was corrected for the droplet path length. For this, an ellipsoid function was used to account for the variability of the light path length along the laser propagation axis to compute the average thickness of the droplet. For a better visualization of the system, Figure 2a presents 3D views of the laser beam intersecting the droplet, with a 2D planar representation given in Figure 2b. Figure 2c shows an example of the temperature distribution of the droplet and needle, generated using ANSYS Transient Thermal software.

**Figure 2:**
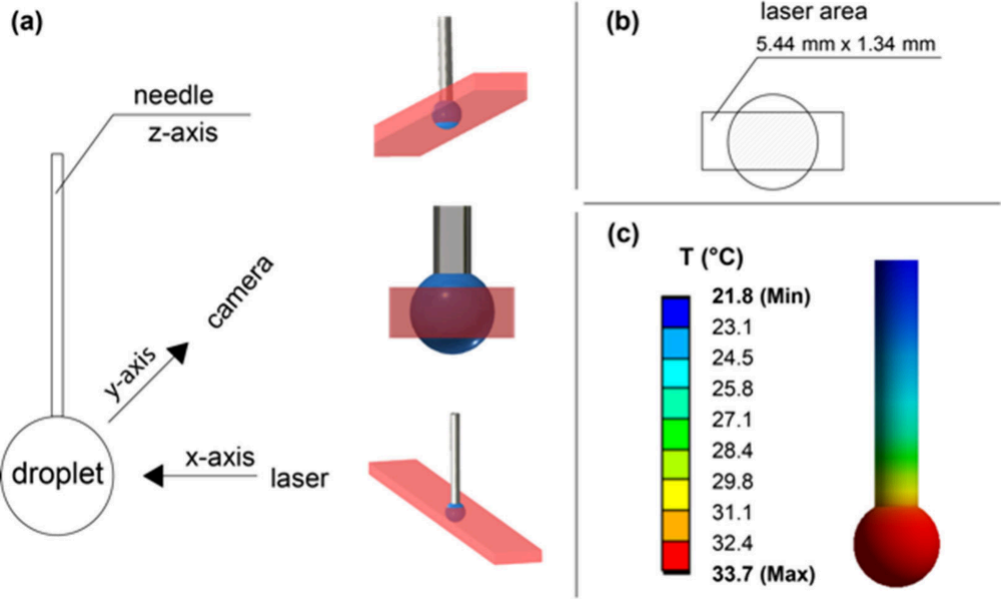
(a) Diagram of the measurement setup and 3D views of the laser area/volume that intersect the droplet. The droplet is in blue and the laser is in red. (b) Planar representation of the laser area that intersects the droplet. (c) Representative droplet-on-needle 3D model with sample temperature distribution.

The nanorod dimensions were 65 ± 9 nm × 16 ± 3 nm (length × diameter). The normalized absorption spectra of the samples are given in Figure 3a, while their morphology is shown in Figure 3b. The absorption maximum was centered at 835 nm for AuNRs and at 842 nm for PEG-AuNRs. The reference sample was distilled water or phosphate buffer saline (PBS), for AuNRs without and with PEG, respectively. A diode laser with a peak wavelength of 808 nm and optical power output of 350 mW was used to irradiate the samples. The incident laser beam power density was measured before each experiment, ranging between 4.3 and 4.4 W cm^–2^, the total incident power was calculated by considering the area of the laser that intersected the droplet, as shown schematically in Figure 2b.

**Figure 3:**
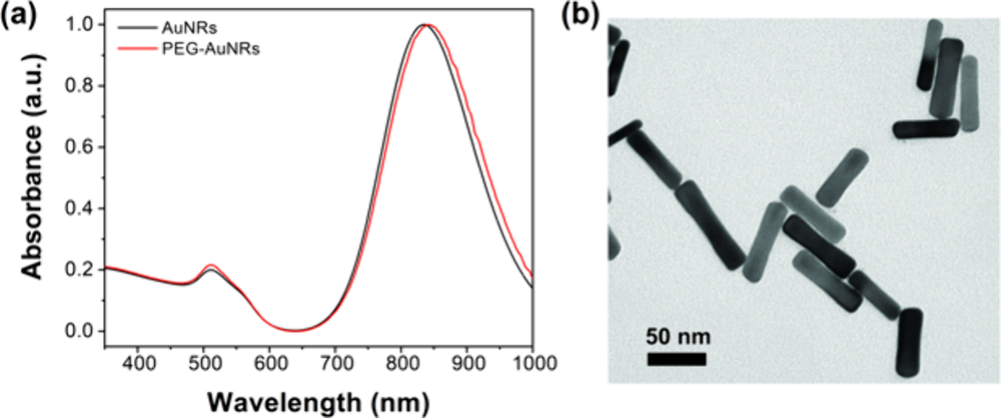
(a) Normalized absorption spectra of AuNRs and PEG-AuNRs. (b) TEM image of AuNRs.

The photothermal conversion efficiency, *η,* was calculated using three independent methods: Roper’s protocol,^10^ Wang’s method,^11^ and numerical analysis with the transient thermal module in ANSYS.

The experiment was conducted three times per sample, for 60 s each (Figure 4a). During the first 30 s, heating was recorded as the droplet was irradiated. Then, the laser was turned off to record the droplet cooling for 30 s. Steady state temperatures were reached within 30 s of heating and cooling, respectively. Thermograms of representative droplets are provided in the insets in Figure 4. The average temperature of the system (which includes the droplet, the sample residing within the needle, and the needle itself) was determined from the thermograms. The thermograms were also used to determine the droplet size based on the proportion between the needle width in pixels and its outer diameter *d*_*n*_ = 1 mm. The relative mass of evaporated droplets corresponded to 14% for 0.25 mM AuNRs, 27% for 0.50 mM AuNRs, and 18% for PEG-AuNRs and was not replenished during the 60 s of measurement time.

**Figure 4:**
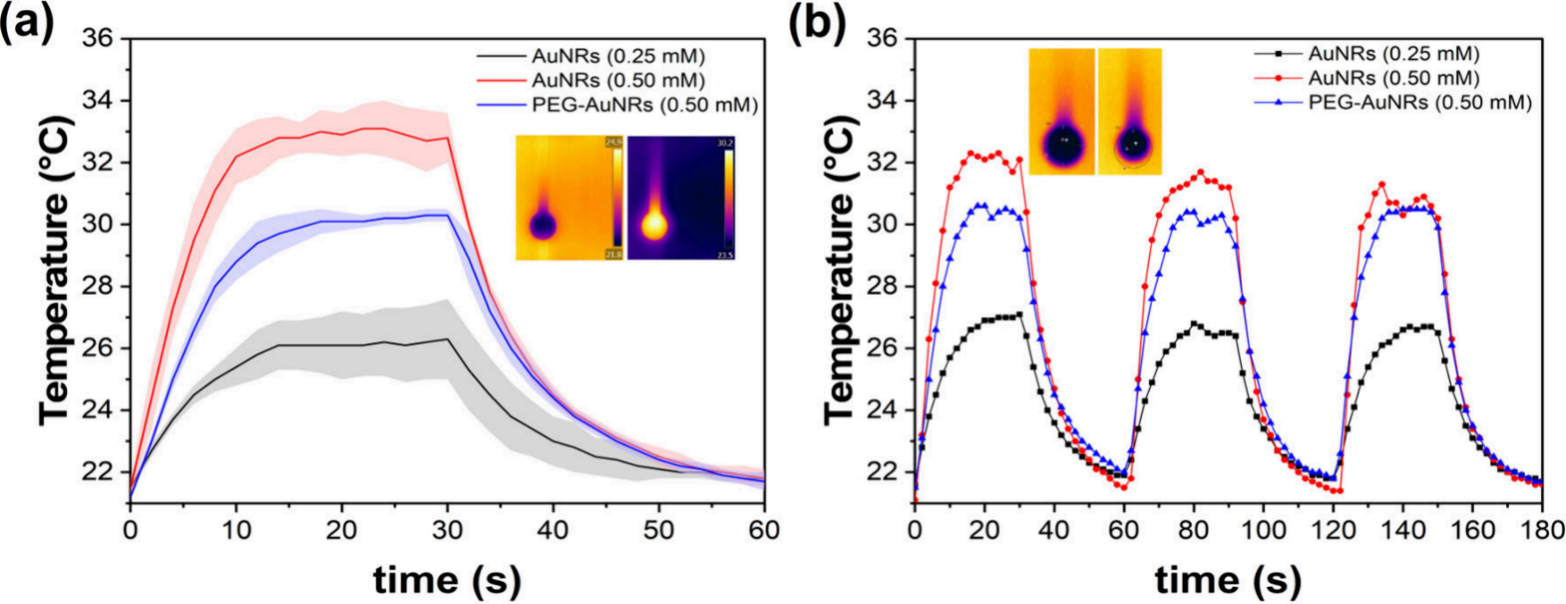
(a) Heating–cooling curves for the droplets of 0.25 mM AuNRs, 0.50 mM AuNRs, and 0.50 mM PEG-AuNRs. The solid lines correspond to the average temperature of three measurements, and the shadows represent the standard deviation. The insets present example thermograms of a droplet under ambient conditions (left) and irradiation (right). (b) ON-OFF cycle evaluation. The insets include a droplet of 0.50 mM Au NRs before (left) and after a 3-cycle experiment (right).

Heating–cooling curves were plotted for each sample (Figure 4a). The highest temperature was achieved with 0.50 mM AuNRs (33.3 ± 0.9 °C); while the lowest was for 0.25 mM AuNRs (26.4 ± 1.1 °C), as expected, due to the lower concentration resulting in a lower absorbance. PEG-AuNRs reached a maximum of 30.3 ± 0.2 °C. This temperature is slightly lower than the one achieved with as-synthesized AuNRs at the same concentration (0.50 mM), which could have resulted from some PEG-AuNRs deposited on the internal walls of the needle decreasing the effective concentration in the droplet.

Three consecutive cycles of heating–cooling were performed for each sample, lasting 180 s in total. Figure 4b depicts a comparison of the performance of the samples throughout three consecutive heating–cooling cycles. The decrease of the maximum temperature at each cycle is due to evaporation. The insets in both Figure 4a and Figure 4b clearly depict the evaporation of the droplet–droplet shrinkage.

Our primary objective was to obtain an accurate estimation of the light-to-heat conversion efficiency, η, for each sample. The results are presented in Figure 5. η, as determined by ANSYS including evaporation, corresponded to 39 ± 6%, 59 ± 4%, and 43 ± 6% for AuNRs at 0.25 mM, 0.50 mM and PEGylated, respectively. These values were similar to the ones calculated through Wang’s approach (*p* >.05). Roper’s method resulted in significant underestimations respective to the ANSYS simulation (*p* <.05). We argue that the most reliable method for determining η is the numerical analysis, primarily due to the fact both Wang’s and Roper’s methods neglect solvent evaporation. In our opinion, both approaches should be updated to accurately account for the transformation of laser thermal energy into solvent evaporation. To test the importance of including or neglecting evaporation, we carried out ANSYS calculations that did not consider evaporation. These calculations returned ∼90% lower η values than simulations that included evaporation. We note Wang’s and Roper’s approaches may indirectly account for the evaporation, plausibly within the time constant or the coefficients *a* and *b*, respectively. We attempted to modify Roper’s and Wang’s methods by including evaporation in the heat balance and calculated η. However, this attempt yielded overestimated and unphysical η values that were equal or higher than 100%.

**Figure 5:**
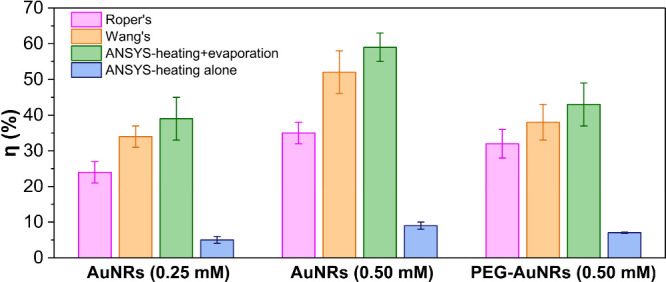
Light-to-heat conversion efficiency (η, %) calculated by three different methods: Roper’s, Wang’s, and ANSYS. The ANSYS approach has been used to compare the effect of evaporation on the estimation of η. In all cases η is presented as the average of three measurements with error bars indicating the standard deviation.

Comparing the performance of different nanotransducers requires unifying the methods for quantifying the photothermal conversion efficiency, as it is an intrinsic property of the material. A useful comparison of different gold nanoheaters is given in Meyer et al.^12^ As the authors state, it is necessary to be careful when comparing reported efficiency values since large discrepancies can be observed even for the same type of nanoparticles. Here, we suggest a simple and more accurate way to calculate η employing numerical simulation methods. These methods enable including different experimental constraints and can be readily extrapolated to many experimental geometries, including cuvette experiments. In case where access to simulation software such as ANSYS is not available, our recommendation is to carry out the measurements in droplets to avoid estimating an “effective mass” and use Wang’s approach to easily receive η values close to the values obtained with ANSYS.

## Supporting Information Available

Detailed information about the heat transfer analysis, synthesis of gold nanorods, PEGylation of gold nanorods, photothermal effect evaluation, and analysis of the thermograms; images of an experiment conducted in a quartz cuvette and experimental setup for the photothermal conversion measurements; Python script used for the numerical determination of coefficients *a* and *b* for Wang’s method; the collected raw data, calculations, and equations not part of the main text; and literature thermodynamic properties (PDF)

## Abbreviations

AuNRs: gold nanorods
PEG: polyethylene glycol
