# Supplementary material for: Numerical Simulation of Light to Heat Conversion by Plasmonic Nanoheaters
Source: Nano Lett. 2024 Dec 19;25(1):230–5. doi: 10.1021/acs.nanolett.4c04872 (PMC11719628; doi:10.1021/acs.nanolett.4c04872)
Supplement: Supplementary file 1 — nl4c04872_si_001.pdf [file nl4c04872_si_001.pdf]

SUPPORTING INFORMATION

Numerical simulation of light to heat conversion by plasmonic nanoheaters

María C. Nevárez Martínez [a, e, *], *Dominik Kreft* [b], *Maciej Grzegorczyk* [c], *Sebastian Mahlik* [c], *Magdalena Narajczyk* [d], *Adriana Zaleska-Medynska* [a], *Demosthenes P. Morales* [e, *], *Jennifer A. Hollingsworth* [e], *James H. Werner* [e]

[a]Department of Environmental Technology, Faculty of Chemistry, University of Gdańsk, Wita Stwosza 63, 80-308 Gdańsk, Poland

[b]Institute of Naval Architecture, Faculty of Mechanical Engineering and Ship Technology, Gdańsk University of Technology, Gabriela Narutowicza 11/12, 80-233 Gdańsk, Poland

[c]Institute of Experimental Physics, Faculty of Mathematics, Physics, and Informatics, University of Gdańsk, Wita Stwosza 57, 80-308 Gdańsk, Poland

[d]Bioimaging Laboratory, Faculty of Biology, University of Gdańsk, Wita Stwosza 59, 80-308 Gdańsk, Poland

[e]Center for Integrated Nanotechnologies, Los Alamos National Laboratory, Los Alamos, 87545, New Mexico, United States of America

* maria_nevarez@lanl.gov; dmorales@lanl.gov

Heat transfer analysis

Energy balance:

All methods start from the energy balance equation, Eq. S1:

$$\sum m_i \cdot c_{p_i} \frac{dT}{dt} = Q_L + Q_O - Q_{ext} \qquad \text{[Eq. S1]}$$

Where $\sum m_i \cdot c_{p_i}$ is the sum of the products of mass times the heat capacity of each system component, $\frac{dT}{dt}$ represents the rate of temperature change. Q_L is the heat transduced by the nanoparticles upon light irradiation while Q_O is the transduced heat after irradiation of the solvent, which was determined to be negligible for distilled water and for PBS, experimentally. Q_{ext} is the heat dissipated to the environment. Q_L and Q_{ext} are expanded in Eq. S2 and Eq. S3, respectively.

$$Q_L = I(1 - 10^{A_\lambda})\eta \qquad \text{[Eq. S2]}$$

$$Q_{ext} = hA_{area}(T_{max} - T_{amb}) \qquad \text{[Eq. S3]}$$

Where I is the laser power (in W, measured with a power meter), A_λ is the absorbance of the sample at the laser irradiation wavelength λ (measured experimentally with a spectrophotometer or derived from Beer-Lambert law). h is the heat transfer coefficient, A_{area} is the heat transfer area to the surroundings, T_{amb} is the ambient air temperature and T_{max} is the maximum temperature reached at steady state.

Roper's method:

According to Roper's [1] approach, it is possible to approximate the product hA_{area} to the $\sum m_i \cdot c_{p_i}$ divided by the cooling time constant, τ_c. When the system reaches the equilibrium (steady state), $\frac{dT}{dt} = 0$ and by substituting the above expressions into Eq. S1, η can be determined from Eq. S4. τ_c is determined from the cooling curve (temperature, *T*, *vs.* time, *t*), when the laser irradiation has stopped, by fitting the collected data to Eq. S5 or a linearized form of it. θ is a dimensionless driving force temperature (Eq. S6).

$$\eta = \frac{\sum m_i \cdot c_{p_i} (T_{max} - T_{amb}) - Q_0}{\tau_c I(1 - 10^{-A_\lambda})} \quad \text{[Eq. S4]}$$

$$\theta = 1 - \exp\left(-\frac{t}{\tau_c}\right) \quad \text{[Eq. S5]}$$

$$\theta = \frac{T_{amb} - T}{T_{amb} - T_{max}} \quad \text{[Eq. S6]}$$

Wang's method:

Wang's approach [2], on the other hand, converts the heat balance equation (Eq. S1) into a descriptive function by introducing the coefficients a and b as in Eq. S7:

$$T(t) = T_0 + \frac{a}{b}(1 - e^{-bt}) \quad \text{[Eq. S7]}$$

Where $T(t)$ is temperature as function of time *t*, and T_0 is the temperature at $t = 0$. The coefficients a and b, were calculated numerically by fitting the temperature curve to the data using a Python script. Then, the photothermal conversion efficiency is estimated from Eq. S8.

$$\eta = \frac{a \cdot \sum m_i \cdot c_{p_i}}{I(1 - 10^{-A_\lambda})} \quad \text{[Eq. S8]}$$

For our system, *m* in Eq. S8 is the droplet/ needle/ water-in-needle mass and c_p is the average specific heat.

Gold nanorod synthesis

Materials: (1-Hexadecyl)trimethylammonium bromide (CTAB, 98%, lot 1392212) was purchased from abcr GmbH, Germany. Silver nitrate ($AgNO_3$, 99.995% trace metal basis, lot MKCN7884), L-ascorbic acid (AA, 99%, lot BCCF9931), sodium borohydride ($NaBH_4$, 99 % reagent plus, lot STBJ7948) and gold(III) chloride trihydrate ($HAuCl_4 \times 3H_2O$, 99.9% trace metal basis, lot MKCP3108) were acquired from Merck Life Science Sp. z o.o. Hydrochloric acid (HCl, 35-37%) was purchased from P. P. H. "STANLAB" Sp. z o.o. All reagents were used as received without further purification. All aqueous solutions were prepared with MilliQ water with conductivity of 0.05 μS.

Considerations: All the used glassware was cleaned with aqua regia made by mixing concentrated hydrochloric and nitric acids in a (4:1) volumetric proportion. The procedure consisted in treating the glassware for 10 min in aqua regia, followed by three washing cycles with DI water in a bath sonicator for 3 min each.

The entire procedure was carried out in a water bath to keep the temperature constant at 28 ±2 °C. The mixtures containing CTAB were gently handled to avoid foam production. Every time, after the addition of the gold precursor to the CTAB solution, it was necessary to mix until no signs of turbidity (orange clots) were present.

Procedure: One large batch sharing the same optical properties was synthesized by the seed-growth method as described by Scarabelli, *et al.* [3] with slight modifications, such as scaling up by a 20 fold. Briefly, the growth solution was prepared by adding 2 mL of 50 mM $HAuCl_4 \times 3H_2O$ to a glass bottle containing 200 mL of 100 mM CTAB. After ensuring full dissolution, the following reagents were dosed consecutively, and in between each addition, the system was thoroughly mixed by sonication avoiding foam formation: 3.8 mL 1 M HCl, 2.4 mL 10 mM

$AgNO_3$, and 2 mL 100 mM L-ascorbic acid (AA). The solution was set aside, in a water bath at 28 ±2 °C until the seed solution was ready for usage.

The seed solution was made by adding 25 µL of 50 mM $HAuCl_4 \times 3H_2O$ to a 15 mL vial containing 4.7 mL of 100 mM CTAB. The solution was mixed until a clear yellow color was observed. The stirring speed was set at 800 rpm, then 300 µL of a freshly prepared ice-cold 10 mM $NaBH_4$ solution was injected. The solution was left to rest without stirring for 5 min in the water bath. Next, 480 µL of seed solution was injected into the above growth solution, which was mixed for 5 min by sonication and then, left undisturbed in the water bath at 28 ±2 °C, for 120 min, while a brown-reddish color developed. The as-obtained gold nanorods (AuNRs) were washed three times in a Sigma Polygen 3-18 k centrifuge at 25 °C and 10 000 RCF until reaching a CTAB concentration of 1 mM. The resulting product was characterized and stored at 4 °C for several months without observing sedimentation or variations in its optical properties.

PEGylation of gold nanorods (PEG-AuNRs)

500 nmol of AuNRs (as Au^0 determined from the absorbance at 400 nm) was added to a 1.5 mL microcentrifuge tube. Then, poly(ethylene glycol) methyl ether thiol (mPEG-SH, 2000 Da, Merck, 1 mM prepared in 0.1 M phosphate buffer (PB) pH 6) was given in 0.1:1 molar ratio mPEG-SH : AuNRs. 100 µL of PB pH 6 was added before continuing to dilute the system e.g., final concentration of 2.7 mM Au^0 in a final 186 µL. The samples were sonicated for 15 min in a bath sonicator and incubated for 2 h at 37 °C and 400 rpm in an Eppendorf ThermoMixer. After that, the sample was washed twice with PB pH 6, with an Eppendorf 5424R centrifuge at 14 000 RCF for 20 min at 4 °C. Finally, the precipitate was redispersed in 1 mL of phosphate buffered saline (PBS, 1×, 137 mM NaCl, 2.7 mM KCl, and 10 mM phosphate pH 7.4).

Photothermal effect evaluation

The experiments were carried out in a lab-made hanging-drop setup (**Figure S2**), which controlled the release of a droplet from a 1 mL KD-Ject syringe with Fisnar 1/2” flexible epoxy seal, and a gauge 20 needle made of PTFE. A RLDH808M-350-5 diode laser manufactured by Roithner LaserTechnik GmbH was used. The laser supports TTL modulation, has a peak wavelength at 808 nm, and has a 350 mW optical output power.

Solutions of AuNRs (0.25 and 0.50 mM) and PEG-AuNRs (0.50 mM) were exposed to light for 30 s. The power was measured with a standard photodiode power sensor (S120VC, UV-extended Si, 200-1100 nm, 50 nW-50 mW, Thorlabs). The temperature was recorded in real time using a FLIR A35 FOV 13 thermal camera (60 Hz, 320 × 256 resolution and ± 5 °C or 5 % accuracy) during the heating (light ON) and cooling processes (light OFF). The camera was connected to the computer using FLIR Thermal Studio software. Three methodologies were applied to calculate the light-to-heat conversion efficiency (η): Roper’s, Wang’s, and modeling in ANSYS software. ANSYS 2021 R2 was used with two Finite Elements Methods modules – Steady State Thermal and Transient Thermal. All modeled bodies, such as sample droplets, needles, and sample in the needles, were considered solid (no convection inside water). The dependent variable was the heat flux, and the primary independent value was the time needed for the droplet temperature to reach a steady state (30 s). Another independent value was the boundary conditions, such as heat transfer to air, needle heat transfer to the syringe body, and radiation to the surroundings.

The η values obtained by the three methods were compared by applying two-tailed t-tests.

***Analysis of the thermograms*:**

The thermographic images were processed using the IR camera software (FLIR Thermal Studio Suite). Each droplet was marked using an ROI in the shape of an ellipse, which covered the droplet.

The temperature recorded for the heating-cooling curves corresponded to the average temperature of the droplet as read from the ellipsoidal ROI, at a given experimental time.

The thermograms were also used to determine the droplet size–ellipsoidal ROI size–based on the proportion of pixels between the needle width and the outer diameter d_n = 1 mm.

***Assumptions and considerations for the simulation*:**

Simplifications were considered for the simulation. The droplets were considered of the same size–average dimensions–throughout the 60 s experiment.

A finite element module divided the droplet into elements that remained in the same position throughout the simulation.

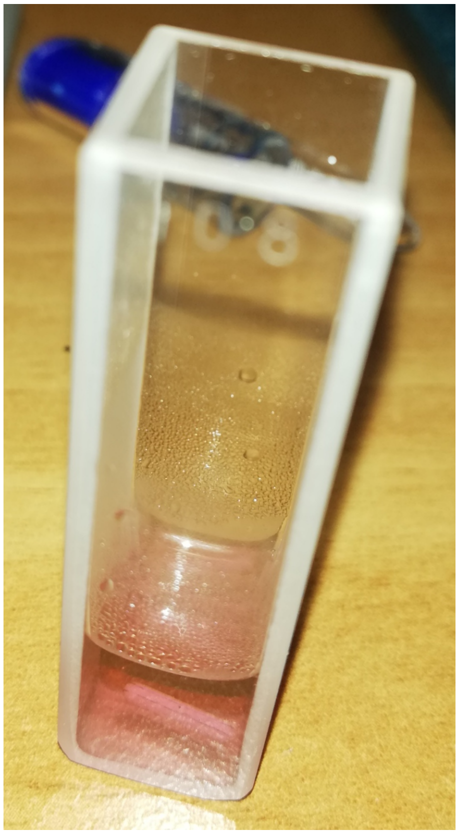

Figure S1. Cuvette with a 1 mL sample of AuNRs that had undergone evaporation and condensation during a photothermal conversion evaluation experiment carried out with an 808 nm laser for 45 min.

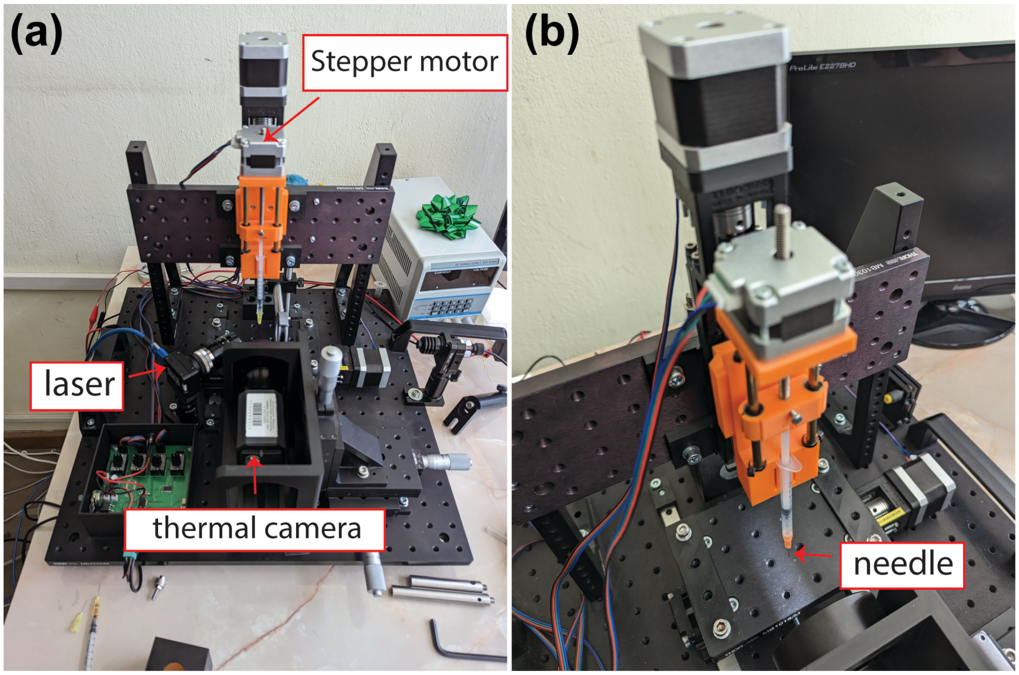

Figure S2. Picture of (a) the full setup for photothermal measurements and (b) closeup for the syringe pump to control the generation of droplets

Python script for the calculation of the coefficients a and b from Wang's method

```
import numpy as np
from scipy.optimize import curve_fit

# Data
t = np.array([0, 2, 4, 6, 8, 10, 12, 14, 16, 18, 20, 22, 24, 26,
28, 30])
T = np.array([21.1, 23.2, 26.3, 28.1, 29.8, 31.2, 31.5, 32,
32.3, 32.2, 32.1, 32.2, 32.3, 32, 31.7, 32.1])

# Function to fit
def model_function(t, a, b):
    T0 = 21.1
    return T0 + a / b * (1 - np.exp(-b * t))

# “a” and “b” parameter fitting to data
params, covariance = curve_fit(model_function, t, T)

# Results
a, b = params
print("a =", a)
print("b =", b)
```

Temperature recorded during the photothermal conversion evaluation

Table S1. Raw temperature data for three repetitions of photothermal conversion

	AuNRs (0.25 mM)			AuNRs (0.50 mM)			PEG-AuNRs (0.50 mM)		
	Temperature (°C)			Temperature (°C)			Temperature (°C)		
time (s)	rep 1	rep 2	rep 3	rep 1	rep 2	rep 3	rep 1	rep 2	rep 3
0	21.5	21.5	21.6	21.6	21.7	21.1	21.3	21.1	21.2
2	22.8	22.4	22.8	25.1	24.9	23.2	23.2	22.9	23.0
4	23.9	23.4	23.8	27.9	27.8	26.3	25.3	24.8	24.8
6	24.7	24.2	24.5	30.1	30.3	28.1	27.1	26.3	26.4
8	25.3	24.6	25.2	31.9	31.6	29.8	28.5	27.6	27.9
10	25.9	24.7	25.7	33.0	32.5	31.2	29.4	28.3	28.8
12	26.4	24.9	26.0	33.1	32.9	31.5	30.2	29.0	29.1
14	26.7	25.2	26.3	33.2	33.3	32.0	30.3	29.1	29.6
16	26.5	25.1	26.6	32.9	33.3	32.3	30.4	29.4	30.0
18	26.5	25.0	26.7	33.2	33.5	32.2	30.4	29.7	30.2
20	26.5	24.8	26.9	33.4	33.3	32.1	30.3	29.8	30.2
22	26.5	25.0	26.9	33.8	33.2	32.2	30.3	29.9	30.2
24	26.7	25.0	27.0	34.0	33.0	32.3	30.3	29.9	30.3
26	26.7	24.7	27.0	33.8	32.9	32.0	30.3	29.9	30.3
28	26.9	24.8	27.0	33.7	32.8	31.7	30.4	30.0	30.4
30	27.0	24.8	27.1	33.6	32.6	32.1	30.4	30.0	30.4
32	25.7	23.8	26.4	29.8	29.9	30.4	29.1	28.0	29.5
34	24.9	23.2	25.4	27.6	27.8	28.1	27.5	26.5	27.5
36	24.3	22.6	24.6	25.9	26.6	26.6	26.2	25.5	26.4
38	23.8	22.3	24.0	24.9	25.5	25.6	25.2	24.7	25.5
40	23.4	22.1	23.6	24.2	24.7	24.7	24.5	24.1	24.6
42	23.2	21.9	23.2	23.7	24.1	23.9	24.0	23.6	23.9
44	22.8	21.8	22.9	23.3	23.7	23.4	23.5	23.2	23.6
46	22.7	21.7	22.7	22.9	23.3	23.0	23.1	22.8	23.2
48	22.5	21.7	22.5	22.6	23.0	22.7	22.7	22.6	22.9
50	22.2	21.7	22.3	22.3	22.8	22.4	22.4	22.3	22.5
52	22.1	21.8	22.2	22.2	22.6	22.1	22.3	22.0	22.2
54	22.0	21.8	22.1	22.0	22.4	22.0	22.2	21.9	22.1
56	22.0	21.7	22.0	22.0	22.1	21.8	22.1	21.7	21.9
58	21.9	21.7	21.9	21.9	22.1	21.6	22.0	21.6	21.8
60	21.8	21.6	21.9	21.8	22.0	21.5	22.0	21.5	21.7

Table S2. Thermodynamic properties and partial calculations for each analyzed droplet

	AuNRs (0.25 mM)			AuNRs (0.50 mM)			PEG-AuNRs (0.50 mM)		
	rep 1	rep 2	rep 3	rep 1	rep 2	rep 3	rep 1	rep 2	rep 3
laser power density (W cm^{-2})	4.3	4.3	4.4	4.4	4.3	4.3	4.4	4.4	4.4
τ_c (s)	7.85	7.81	7.30	6.42	6.75	7.56	6.07	7.65	7.34
initial droplet mass (mg)	7.14	7.89	7.06	4.10	4.67	5.02	6.22	5.85	4.62
evaporated droplet mass (mg)	0.93	1.20	0.90	1.14	1.36	1.27	1.04	0.80	1.06
water density (kg m^{-3})	997								
water heat capacity (J kg^{-1} K^{-1})	4182	4183	4182	4182	4181	4182	4182	4182	4182
latent heat of evaporation (kJ kg^{-1})	2446	2447	2446	2443	2442	2442	2443	2444	2443
"a" from Wang's method	0.854	0.854	0.868	2.470	2.519	1.958	1.580	1.328	1.344
"b" from Wang's method	0.158	0.195	0.143	0.202	0.217	0.172	0.170	0.145	0.141
$\dot{Q}_{\Delta T}$ from ANSYS (W cm^{-3})	0.87	0.58	0.84	2.30	2.02	1.97	1.40	1.38	1.67

REFERENCES

(1) Roper, D. K.; Ahn, W.; Hoepfner, M. Microscale heat transfer transduced by surface plasmon resonant gold nanoparticles. *The Journal of Physical Chemistry C* **2007**, *111* (9), 3636-3641. DOI: 10.1021/jp064341w.

(2) Wang, X.; Li, G.; Ding, Y.; Sun, S. Understanding the photothermal effect of gold nanostars and nanorods for biomedical applications. *RSC Adv.* **2014**, *4* (57), 30375-30383. DOI: 10.1039/c4ra02978j.

(3) Scarabelli, L.; Sánchez-Iglesias, A.; Pérez-Juste, J.; Liz-Marzán, L. M. A "tips and tricks" practical guide to the synthesis of gold nanorods. *The Journal of Physical Chemistry Letters* **2015**, *6* (21), 4270-4279. DOI: 10.1021/acs.jpclett.5b02123.
